# Adjunctive immunotherapeutic agents in patients with sepsis and septic shock: a multidisciplinary consensus of 23

**DOI:** 10.1186/s44158-024-00165-3

**Published:** 2024-04-30

**Authors:** Massimo Girardis, Irene Coloretti, Massimo Antonelli, Giorgio Berlot, Stefano Busani, Andrea Cortegiani, Gennaro De Pascale, Francesco Giuseppe De Rosa, Silvia De Rosa, Katia Donadello, Abele Donati, Francesco Forfori, Maddalena Giannella, Giacomo Grasselli, Giorgia Montrucchio, Alessandra Oliva, Daniela Pasero, Ornella Piazza, Stefano Romagnoli, Carlo Tascini, Bruno Viaggi, Mario Tumbarello, Pierluigi Viale

**Affiliations:** 1 Anesthesia and Intensive Care Medicine, Policlinico Di Modena, University of Modena and Reggio Emilia, Modena, Italy; 2 Dipartimento Di Scienze Biotecnologiche Di Base, Cliniche Intensivologiche E Perioperatorie, Università Cattolica del Sacro Cuore, Rome, Italy; 3 Dipartimento Di Scienze Dell’Emergenza, Anestesiologiche E Della Rianimazione, Fondazione Policlinico Universitario A. Gemelli IRCCS, Rome, Italy; 4 Anesthesia and Intensive Care, Azienda Sanitaria Universitaria Giuliano Isontina, Trieste, Italy; 5 Department of Surgical, Oncological and Oral Science (Di.Chir.On.S.), University of Palermo, Palermo, Italy; 6 Department of Anaesthesia, Intensive Care and Emergency, Policlinico Paolo Giaccone, Palermo, Italy; 7 Unit of Infectious Diseases, Cardinal Massaia Hospital, Asti, Italy; 8 Anesthesia and Intensive Care, Santa Chiara Regional Hospital, APSS, Trento, Italy; 9 Department of Surgery, Dentistry, Ginaecology and Paediatrics, University of Verona, and Anesthesia and Intensive Care Unit B, University Hospital Integrated Trust of Verona, Verona, Italy; 10 Anesthesia and Intensive Care, Azienda Ospedaliero Universitaria Delle Marche, Ancona, Italy; 11 Anesthesia and Intensive Care, Anesthesia and Resuscitation Department, Azienda Ospedaliero Universitaria Pisana, Pisa, Italy; 12 Department of Medical and Surgical Sciences Infectious Diseases Unit, IRCCS Azienda Ospedaliero Universitaria Di Bologna, Alma Mater Studiorum University of Bologna, Bologna, Italy; 13 Department of Medical and Surgical Sciences, Alma Mater Studiorum University of Bologna, Bologna, Italy; 14 Department of Anesthesia, Intensive Care and Emergency, Fondazione IRCCS Ca’ Granda Ospedale Maggiore Policlinico, Milan, Italy; 15 Department of Pathophysiology and Transplantation, University of Milan, Milan, Italy; 16 Department of Surgical Sciences, Departement of Anesthesia, Resuscitation and Emergency Torino, University of Turin, Turin, Italy; 17 Department of Public Health and Infectious Diseases, Sapienza University of Rome, Rome, Italy; 18 Department of Medicine, Surgery and Pharmacy, University of Sassari, Sassari, Italy; 19 University Hospital “San Giovanni Di Dio E Ruggi d’Aragona”, Salerno, Italy; 20 Department of Health Science, Department of Anesthesia and Intensive Care, University of Florence, Careggi University Hospital, Florence, Italy; 21 Department of Medicine (DAME), Infectious Diseases Clinic, University of Udine, Udine, Italy; 22 Anesthesia and Intensive Care, Careggi University Hospital, Florence, Italy; 23 Infectious and Tropical Diseases Unit, Azienda Ospedaliera Universitaria Senese, Siena, Italy

**Keywords:** Sepsis, Septic shock, Adjunctive therapies, Corticosteroids, Immunoglobulins, Blood purification, Checkpoint immune therapies, Specific immune therapies

## Abstract

**Background:**

In the last decades, several adjunctive treatments have been proposed to reduce mortality in septic shock patients. Unfortunately, mortality due to sepsis and septic shock remains elevated and NO trials evaluating adjunctive therapies were able to demonstrate any clear benefit. In light of the lack of evidence and conflicting results from previous studies, in this multidisciplinary consensus, the authors considered the rational, recent investigations and potential clinical benefits of targeted adjunctive therapies.

**Methods:**

A panel of multidisciplinary experts defined clinical phenotypes, treatments and outcomes of greater interest in the field of adjunctive therapies for sepsis and septic shock. After an extensive systematic literature review, the appropriateness of each treatment for each clinical phenotype was determined using the modified RAND/UCLA appropriateness method.

**Results:**

The consensus identified two distinct clinical phenotypes: patients with overwhelming shock and patients with immune paralysis. Six different adjunctive treatments were considered the most frequently used and promising: (i) corticosteroids, (ii) blood purification, (iii) immunoglobulins, (iv) granulocyte/monocyte colony-stimulating factor and (v) specific immune therapy (i.e. interferon-gamma, IL7 and AntiPD1). Agreement was achieved in 70% of the 25 clinical questions.

**Conclusions:**

Although clinical evidence is lacking, adjunctive therapies are often employed in the treatment of sepsis. To address this gap in knowledge, a panel of national experts has provided a structured consensus on the appropriate use of these treatments in clinical practice.

**Supplementary Information:**

The online version contains supplementary material available at 10.1186/s44158-024-00165-3.

## Background

In the last decade, sepsis and septic shock have shown a continuously growing incidence and persistently elevated mortality rates, higher than 20% for sepsis and 50% for septic shock, despite general improvements in the application of specific treatment protocols [1–3]. To further reduce mortality associated with sepsis, several adjunctive treatments have been proposed, particularly for more complicated patients. Unfortunately, due to the negative results of several randomised trials, the use of these adjunctive therapies is not recommended in more recent evidence-based guidelines [4]. The exploration of pathobiological mechanisms has uncovered a remarkable diversity of inflammatory responses in sepsis. In addition to the most common clinical presentation, which is characterised by a sudden, dysregulated, pro-inflammatory reaction featuring fever, vasodilation and hyperdynamic circulation, a distinct response may manifest in earlier or later stages as a blunted pro-inflammatory phase. The prevalence of immunosuppressive mechanisms corresponds to various clinical phenotypes characterised by the persistence of organ dysfunction and sepsis progression, as well as the occurrence of secondary opportunistic infections. This extensive heterogeneity of inflammatory responses in sepsis patients may, in part, account for the disappointing outcomes of large randomised controlled trials on adjunctive treatments. In the future, assessment of immune responses using specific biomarkers may enable the design of more precise clinical trials that could include a more homogeneous population of patients with sepsis, allowing a more focused evaluation of the potential clinical benefits of targeted adjunctive therapies.

In recent years, a plea has arisen from the scientific community for the personalisation of therapies in patients with sepsis based on identifiable phenotypes or immunotypes, despite the lack of evidence [5]. To address this need, a multidisciplinary consensus of experts was established to evaluate the available literature and share ideas and experiences on the potential role of the most commonly used and promising adjunctive therapies in specific phenotypes of patients. The consensus identified two distinct clinical scenarios: patients with overwhelming shock from community-acquired infections, and patients with hospital-acquired infections and immune paralysis. This study presents the results of a structured consensus procedure from a multidisciplinary working group of experts from a single high-income country.

## Methods

Two chairs, MG and PV, proposed the formation of a multidisciplinary panel of 20 experts in the fields of intensive care medicine and infectious diseases. All of these experts had a minimum of 10 years of clinical experience in managing patients with sepsis, prominent research profiles and active participation in national and international scientific societies, making them some of the most respected experts in the field of sepsis and infections in Italy.

In the first structured meeting, after an initial discussion, the panellists defined the populations, treatments and outcomes of greater interest in the field of adjunctive therapies in sepsis and agreed on the methods for consensus.

Two different populations were identified: (i) patients admitted to the intensive care unit (ICU) with sepsis or septic shock with an abrupt and dysregulated hyperinflammatory response due to community-acquired infections (usually caused by non-MDR microorganisms), such as invasive pneumococcal and meningococcal diseases, NSTI and streptococcal toxic shock syndrome; and ii) patients admitted to the ICU with sepsis or septic shock and suspected immune dysfunction/immune paralysis, such as late ventilator-associated pneumonia, Candida spp. peritonitis, or bacteraemia caused by opportunistic agents. The panel selected six adjunctive treatments: (i) corticosteroids, (ii) blood purification, (iii) immunoglobulins, (iv) granulocyte/monocyte colony-stimulating factor and (v) specific immune therapy such as interferon-gamma, IL7 and Anti-PD1. ICU, hospital and overall mortality; shock duration; mechanical ventilation; ICU stay; hospital stay; and rate of reinfection were selected as relevant outcomes. Owing to the contrasting and low-quality evidence available, the panellists decided to use a modified semiquantitative RAND/UCLA appropriateness method [6]. This semiquantitative approach allows each component of the panel to express an opinion that is not influenced by other experts and compensates for the lack of evidence regarding the experience and personal opinion of the panellists.

After the first meeting, a systematic review of the literature was performed by one of the authors (IC) using three electronic databases: PubMed, EMBASE and Cochrane Library. All literature materials were readily available at any time for all panellists. For each group of patients and therapy, two individuals on the review panel examined the relevant literature, created a standardised summary of the data (refer to the Supplementary material), and subsequently formulated the official questions that were subject to the final vote. This material was presented to other panellists during a second structured meeting held 3 months later. During this meeting, the literature data were reviewed and discussed by the whole group, and if any controversies occurred, the list of statements was better redefined to avoid uncertainties in the rating procedures.

For the final anonymous vote, we used the RAND/UCLA method on an online platform. The appropriateness of each treatment in each scenario was rated by all panellists on a scale of 1 to 9, with 1 = always inappropriate and 9 = always appropriate. Treatment indications were classified based on the median as ‘appropriate’ (median 7–9), ‘inappropriate’ (median 1–3) or ‘uncertain’ (median 4–6). ‘Disagreement’ for each treatment indication in each scenario was calculated using the IPRAS method developed by BIOMED Concerted Action on Appropriateness [6]. After the first round, the group results were reported individually to each panellist, who in the second rating round could either confirm or modify their previous choice. No further scoring rounds were conducted. When disagreement was confirmed in the second round, the indication will be ‘Uncertain’ regardless of the rate achieved.

It is important to recognise that the chairs and panellists of the consensus process are all from a single high-income country, which significantly limits the ability to generalise the results to other settings and countries, particularly those with different income levels.

### Scenario 1. Patient with sepsis and septic shock due to community-acquired infections with abrupt and dysregulated hyperinflammatory response

#### Description of scenario

**Fig. 1:**
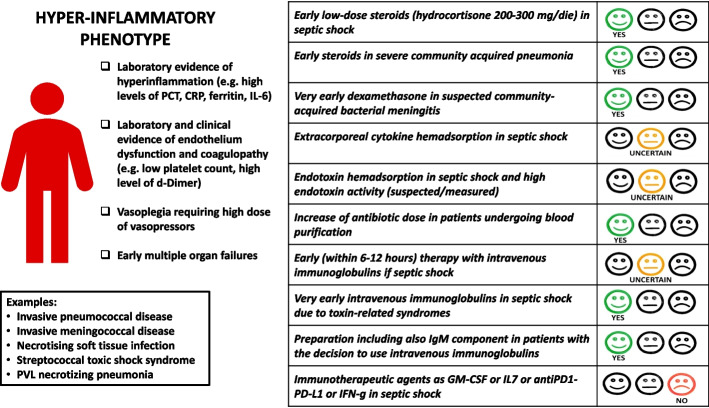
Hyperinflammatory phenotype

In the early phases of sepsis, the pro-inflammatory response often predominates, and its phylogenetic goal is the eradication of pathogens. This phase is characterised by the massive production of proinflammatory cytokines such as tumour necrosis factor (TNF)-alpha, IL-1b, IL-6 and IFN-γ, which stimulate the effector functions of neutrophils, macrophages and Th1 cells by enhancing cellular immunity [7]. Dysregulation of these mechanisms, associated with an inappropriate anti-inflammatory response, may result in multiple organ dysfunction, overwhelming shock and death [8, 9]. During this phase, functional impairment of the endothelium plays a key role in inducing a sudden and protracted state of vasoplegia, increased vascular permeability and activation of the extrinsic pathway of coagulation, resulting in a hypercoagulable state and disseminated intravascular coagulopathy [9–11]. Moreover, several cytokines have direct toxic effects on cardiomyocytes, causing myocardial depression [12]. The archetypes of a dysregulated hyperinflammatory response are usually clinical conditions related to community-acquired infections such as invasive pneumococcal or meningococcal diseases, necrotising soft tissue infections and streptococcal toxic shock syndrome. The hyperinflammatory scenario refers to a previously healthy patient who develops a community-acquired infection that triggers an aberrant immune response with a sudden occurrence of organ failure and vasoplegia resistant to high doses of vasopressors associated to laboratory evidence of hyperinflammation (e.g. high levels of procalcitonin, C-reactive protein, ferritin) and coagulopathy (e.g. low platelet count, high level of d-Dimer) (Fig. 1).

#### Adjunctive therapies (Table 1)

**Table 1:** Questions and results of the ballot for hyperinflammatory phenotype

STEROIDS	
1. How appropriate is, in selected patients with refractory septic shock and severe hyperinflammatory response, the early (within 4–6 h) use of low-dose steroids (i.e. hydrocortisone 200–300 mg/day)?	***APPROPRIATE*** *Median score 8 (IQR 8–8)* *Agreement: YES*
2. How appropriate is, in patients with septic shock and severe hyperinflammatory response and with the decision to use low-dose steroids (i.e. hydrocortisone 200–300 mg/day), the continuous infusion as opposed to repeated bolus infusion?	***UNCERTAIN*** *Median score 5 (IQR 5–6)* *Agreement: YES*
3. How appropriate is, in patients with refractory septic shock and severe hyperinflammatory response, to withdraw (when initially administered) low-dose steroids therapy (i.e. hydrocortisone 200–300 mg/day) when patients no longer need vasopressors?	***APPROPRIATE*** *Median score 8 (IQR 7–9)* *Agreement: YES*
4. How appropriate is, in patients with severe community-acquired pneumonia, the early use (within 24 h) of steroids (i.e. methylprednisolone 40 mg/day or hydrocortisone 200 mg/day)?	***APPROPRIATE*** *Median score 7 (IQR 6–8)* *Agreement: YES*
5. How appropriate is, in patients with severe community-acquired pneumonia with diagnosis of influenza, the early (within 24 h) use of steroids (i.e. methylprednisolone 40 mg/day or hydrocortisone 200 mg)?	***NOT APPROPRIATE*** *Median score 2 (IQR 1–3)* *Agreement: YES*
6. How appropriate is, in patients with suspected community-acquired bacterial meningitis, a very early (before or concomitant to antibiotic administration) therapy with dexamethasone (0.6 mg/kg/day for 4 days)?	***APPROPRIATE*** *Median score 8 (IQR 8–9)* *Agreement: YES*
**BLOOD PURIFICATION**	
1. How appropriate is, in patients with septic shock and severe hyperinflammatory response, the use of high-volume haemofiltration (HVHF)?	***NOT APPROPRIATE*** *Median score 3 (IQR 1–4)* *Agreement: YES*
2. How appropriate is, in patients with septic shock and severe hyperinflammatory response, the use of extracorporeal cytokine hemadsorption?	***UNCERTAIN*** *Median score 3 (IQR 3–5)* *Agreement: NO*
3. How appropriate is, in patients with septic shock with severe hyperinflammatory response and high endotoxin activity (suspected or measured), the use of endotoxin hemadsorption?	***UNCERTAIN*** *Median score 6 (IQR 5–7)* *Agreement: NO*
4. How appropriate is, in patients with septic shock and severe hyperinflammatory response, the use of Coupled Plasma Filtration Adsorption (CPFA)?	***NOT APPROPRIATE*** *Median score 1 (IQR 1–2)* *Agreement: YES*
5. How appropriate is, in patients with septic shock and severe hyperinflammatory response, the use of a blood purification technique only when used early (within 6–12 h)?	***UNCERTAIN*** *Median score 5 (IQR 3–6)* *Agreement: NO*
6. How appropriate is, in patients with septic shock undergoing blood purification, the increase of antibiotic dose?	***APPROPRIATE*** *Median score 8 (IQR 7–8)* *Agreement: YES*
**IMMUNOGLOBULINS**	
1. How appropriate is, in patients with septic shock and severe hyperinflammatory response, the early (within 6–12 h) therapy with intravenous immunoglobulins?	***UNCERTAIN*** *Median score 6 (IQR 5–7)* *Agreement: NO*
2. How appropriate is, in patients with septic shock and severe hyperinflammatory response due to toxin-related syndromes (e.g. invasive meningococcal diseases, pneumococcal or meningococcal Purpura fulminans, necrotizing fasciitis/TSST, PVL necrotizing pneumonia), the very early therapy (within 6 h) with intravenous immunoglobulins?	***APPROPRIATE*** *Median score 8 (IQR 7–8)* *Agreement: YES*
3. How appropriate is, in patients with septic shock and severe hyperinflammatory response and with the decision to use intravenous immunoglobulins, the use of a preparation including also IgM component?	***APPROPRIATE*** *Median score 8 (IQR 7–9)* *Agreement: YES*
4. How appropriate is, in patients with septic shock and severe hyperinflammatory response due to toxin-related syndromes (e.g. invasive meningococcal diseases, pneumococcal or meningococcal Purpura fulminans, necrotizing fasciitis/TSST, PVL necrotizing pneumonia), the very early therapy (within 1–3 h) with the decision to use intravenous immunoglobulin, the use of a preparation including also IgM component?	***APPROPRIATE*** *Median score 8 (IQR 8–9)* *Agreement: YES*
**OTHER IMMUNOTHERAPEUTIC AGENTS**	
1. How appropriate is, in patients with septic shock and severe hyperinflammatory response, the use of immunotherapeutic agents as GM-CSF?	**NOT APPROPRIATE** *Median score 3 (IQR 2–4)* *Agreement: YES*
2. How appropriate is, in patients with septic shock and severe hyperinflammatory response, the use of immunotherapeutic agents as IL7 or antiPD1-PD-L1 or IFN-g?	**NOT APPROPRIATE** *Median score 2 (IQR 2–4)* *Agreement: YES*

##### Steroids

1. How appropriate is, in patients with refractory septic shock and severe hyperinflammatory response, the early (within 4–6 h) use of low-dose steroids (i.e. hydrocortisone 200–300 mg/day)?
*Consensus Rating* *:*Appropriate*; median score 8 (IQR 8–8); Disagreement: NO*
  All panellists voted in the 7–9 region.
2. How appropriate is, in patients with refractory septic shock and severe hyperinflammatory response and with the decision to use low-dose steroids (i.e. hydrocortisone 200–300 mg/day), the continuous infusion as opposed to repeated bolus infusion? 
*Consensus Rating* *: *Uncertain*; median score 5 (IQR 5-6); Disagreement: NO*
  5.2% voted in the 1–3 region, 89.5% voted in the 4–6 region and 5.2% voted in the 7–9 region
3. How appropriate is, in patients with refractory septic shock and severe hyperinflammatory response, to withdraw (when initially administered) low-dose steroid therapy (i.e. hydrocortisone 200–300 mg/day) when patients no longer need vasopressors?
*Consensus Rating* *: *Appropriate*; median score 8 (IQR 7-9); Disagreement: NO*
  10.5% voted in the 1–3 region, 89.5% voted in the 7–9 region
4. How appropriate is, in patients with severe community-acquired pneumonia, the early use (within 24 h) of steroids (i.e. methylprednisolone 40 mg/day or hydrocortisone 200 mg/day)?
*Consensus Rating* *: *Appropriate*; median score 7 (IQR 6–8); Disagreement: NO*
  26.3% voted in the 4–6 region, 73.7% voted in the 7–9 region
5. How appropriate is, in patients with severe community-acquired pneumonia with diagnosis of influenza, the early (within 24 h) use of steroids (i.e. methylprednisolone 40 mg/day or hydrocortisone 200 mg)?
*Consensus Rating* *: *Not Appropriate*; median score 2 (IQR 1-3); Disagreement: NO*
  89.5% voted in the 1–3 region, 10.5% voted in the 4–6 region
6. How appropriate is, in patients with suspected bacterial meningitis, a very early therapy with dexamethasone (0.6 mg/kg/day or equivalent for 5–7 days)?
*Consensus Rating* *: *Appropriate*; median score 8 (IQR 8–9); Disagreement: NO*
  All panellists voted in the 7–9 region

##### Blood purification

1. How appropriate is, in patients with septic shock and severe hyperinflammatory response, the use of high-volume haemofiltration (HVHF)?
*Consensus Rating* *: *Not Appropriate*; median score 3 (IQR 1–4); Disagreement: NO*
  68.4% voted in the 1–3 region, 31.6% voted in the 4–6 region
2. How appropriate is, in patients with septic shock and severe hyperinflammatory response, the use of extracorporeal cytokine hemadsorption?
*Consensus Rating* *: *Uncertain*; median score 3 (IQR 3–5); Disagreement: YES*
  52.6% voted in the 1–3 region, 31.6% voted in the 4–6 region, 15.8% voted in the 7–9 region
3. How appropriate is, in patients with septic shock with severe hyperinflammatory response and high endotoxin activity (suspected or measured), the use of endotoxin hemadsorption?
*Consensus Rating* *: *Uncertain*; median score 6 (IQR 5–7); Disagreement: YES*
  5.3% voted in the 1–3 region, 57.9% voted in the 4–6 region, 36.8% voted in the 7–9 region
4. How appropriate is, in patients with septic shock and severe hyperinflammatory response, the use of Coupled Plasma Filtration Adsorption (CPFA)?
*Consensus Rating* *: *Not Appropriate*; median score 1 (IQR 1–2); Disagreement: NO*
  All panellists voted in the 1–3 region
5. How appropriate is, in patients with septic shock and severe hyperinflammatory response, the use of a blood purification technique only when used early (within 6–12 h)?
*Consensus Rating* *: *Uncertain*; median score 5 (IQR 3–6); Disagreement: YES*
  26.3% voted in the 1–3 region, 52.6% voted in the 4–6 region, 21.1% voted in the 7–9 region
6. How appropriate is, in patients with septic shock undergoing blood purification, the increase of antibiotic dose?
*Consensus Rating* *: *Appropriate*; median score 8 (IQR 7–8); Disagreement: NO*
  15.8% voted in the 4–6 region, 84.2% voted in the 7–9 region

##### Immunoglobulins

1. How appropriate is, in patients with septic shock and severe hyperinflammatory response, the early (within 6–12 h) therapy with intravenous immunoglobulins?
*Consensus Rating* *: *Uncertain*; median score 6 (IQR 5–7); Disagreement: YES*
  15.7% voted in the 1–3 region, 47.4% voted in the 4–6 region, 36.8% voted in the 7–9 region
2. How appropriate is, in patients with septic shock and severe hyperinflammatory response due to toxin-related syndromes (e.g. invasive meningococcal diseases, pneumococcal or meningococcal Purpura fulminans, NSTI/TSST, PVL necrotizing pneumonia), the very early therapy (within 6 h) with intravenous immunoglobulins?
  *Consensus Rating* *: *Appropriate*; median score 8 (IQR 7–8); Disagreement: NO*
  5.3% voted in the 1–3 region, 5.3% voted in the 4–6 region, 89.4% voted in the 7–9 region
3. How appropriate is, in patients with septic shock and severe hyperinflammatory response and with the decision to use intravenous immunoglobulins, the use of a preparation including also IgM component?
*Consensus Rating* *: *Appropriate*; median score 8 (IQR 7–9); Disagreement: NO*
  5.3% voted in the 1–3 region, 15.8% voted in the 4–6 region, 78.9% voted in the 7–9 region

##### Other immunotherapeutic agents

1. How appropriate is, in patients with septic shock and severe hyperinflammatory response, the use of immunotherapeutic agents as GM-CSF?
*Consensus Rating* *: *Not Appropriate*; median score 3 (IQR 2-4); Disagreement: NO*
  73.7% voted in the 1–3 region, 26.3% voted in the 4–6 region
2. How appropriate is, in patients with septic shock and severe hyperinflammatory response, the use of immunotherapeutic agents as IL7 or antiPD1-PDL1 or IFN-g?
*Consensus Rating* *: *Not Appropriate*; median score 2 (IQR 2–4); Disagreement: NO*
  73.7% voted in the 1–3 region, 26.3% voted in the 4–6 region

### Scenario 2. Patients with sepsis or septic shock due to hospital-acquired infections and suspected immune dysfunction / immune paralysis

#### Description of scenario

**Fig. 2:**
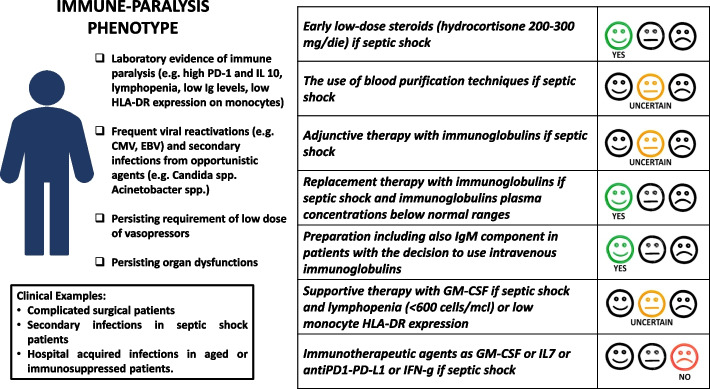
Immune-paralysis phenotype

In sepsis, the anti-inflammatory response mediated by molecules, such as IL-10, IL-4 and TGF-β, is finalised to preserve tissues and mitigate organ damage caused by the initial pro-inflammatory response. However, dysregulated and/or persistent activation of anti-inflammatory mediators/pathways may cause severe failure of the immune system, defined as immune paralysis, characterised by impaired phagocytosis, alteration of cytokine profile, inadequacy of antigen-presenting mechanisms and dysfunction and apoptosis of B and T lymphocytes [13, 14]. Patients with immune paralysis are unable to mount an appropriate inflammatory response and are prone to viral reactivation and secondary or breakthrough infections, mostly caused by opportunistic agents with limited treatment resources, such as Acinetobacter spp. and Candida spp [15, 16]. In contrast to the hyperinflammatory phenotype, mortality in these patients depends on recurrent and persistent infections and usually occurs later, within the second to third week of diagnosis [17–19]. Sepsis or septic shock, in patients with immunoparalysis, might be associated with normo-hypothermia. The elderly population, patients with nosocomial infections, chronic severe comorbidities (e.g. diabetes) and previous immune depression frequently show a blunted inflammatory response and predominant anti-inflammatory pattern [19]. An example of this scenario is a patient of advanced age with persistent anastomotic leaks after abdominal surgery and broad-spectrum antibiotic use, who developed invasive candidiasis. This patient frequently shows a persisting requirement for low doses of vasopressors and not resolving organ dysfunctions associated with laboratory evidence of immune paralysis (e.g. lymphopenia, low Ig levels, low HLA-DR expression on monocytes) (Fig. 2).

In recent years, numerous biomarkers have been proposed to identify patients with immune paralysis; however, most of these biomarkers are not yet ready for bedside use. Nevertheless, some easy-to-measure biomarkers are currently available that may provide a rough but sound indication of the efficiency of the immune response. For instance, HLA-DR expression in monocytes, lymphocyte count, neutrophil-to-lymphocyte ratio and immunoglobulin plasma concentration are closely related to the risk of developing new infections and mortality in different populations of critically ill patients. Similarly, the reactivation of Herpesviridae as well as infection by an opportunistic agent have also been considered reliable and used for identification of an immunosuppressive pattern [16, 20, 21].

#### Adjunctive therapies (Table 2)

**Table 2:** Questions and results of ballot for phenotype with immune paralysis

STEROIDS	
1. How appropriate is, in patients with refractory septic shock and suspected immune dysfunction / immune paralysis, the early (within 4–6 h) use of low-dose steroids (i.e. hydrocortisone 200–300 mg/day)?	***APPROPRIATE*** *Median score 6 (IQR 3–7)* *Agreement: YES*
**BLOOD PURIFICATION**	
1. How appropriate is, in patients with septic shock and suspected immune dysfunction / immune paralysis, the use of a blood purification technique?	***UNCERTAIN*** *Median score 4 (IQR 3–5)* *Agreement: NO*
**IMMUNOGLOBULINS**	
1. How appropriate is, in patients with septic shock and suspected immune dysfunction / immune paralysis, the adjunctive therapy with intravenous immunoglobulins?	***UNCERTAIN*** *Median score 6 (IQR 5–7)* *Agreement: NO*
2. How appropriate is, in patients with septic shock and suspected immune dysfunction/immune paralysis if the plasma concentration of immunoglobulins is below normal ranges, the replacement therapy with intravenous immunoglobulins?	***APPROPRIATE*** *Median score 7 (IQR 6–8)* *Agreement: YES*
3. How appropriate is, in patients with septic shock and suspected immune dysfunction/immune paralysis and with decision to use intravenous immunoglobulins, the use of a preparation including also IgM component?	***APPROPRIATE*** *Median score 8 (IQR 7–9)* *Agreement: YES*
**OTHER IMMUNOTHERAPEUTIC AGENTS**	
1. How appropriate is, in patients with septic shock and suspected immune dysfunction/immune paralysis, and lymphopenia (< 600 cells/mcl) or low monocyte HLA-DR expression, the supportive therapy with GM-CSF?	**UNCERTAIN** *Median score 5 (IQR 4–7)* *Agreement: NO*
2. How appropriate is, in patients with septic shock and suspected immune dysfunction/immune paralysis, the use of other immunotherapeutic agents (i.e. IL7, IFN-gamma, Anti PD1-PDL1)?	**UNCERTAIN** *Median score 4 (IQR 3–5)* *Agreement: NO*

##### Steroids

1. How appropriate is, in patients with refractory septic shock and suspected immune dysfunction / immune paralysis, the early (within 4–6 h) use of low-dose steroids (i.e. hydrocortisone 200–300 mg/day)?
*Consensus Rating* *: *Uncertain*; median score 6 (IQR 3-7); Disagreement: YES*
  26.4% voted in the 1–3 region, 36.8% voted in the 4–6 region, 36.8% voted in the 7–9 region

##### Blood purification

1. How appropriate is, in patients with septic shock and suspected immune dysfunction / immune paralysis, the use of a blood purification technique?
  *Consensus Rating: *Uncertain*; median score 4 (IQR 3–5); Disagreement: YES*
  42.1% *voted in the 1–3 region, 57.9% voted in the 4–6 region*

##### Immunoglobulins

1. How appropriate is, in patients with septic shock and suspected immune dysfunction / immune paralysis, the adjunctive therapy with intravenous immunoglobulins?
*Consensus Rating* *: *Uncertain*; median score 6 (IQR 5-7); Disagreement: YES*
  10.5% voted in the 1–3 region, 47.4% voted in the 4–6 region, 42.1% voted in the 7–9 region
2. How appropriate is, in patients with septic shock and suspected immune dysfunction/immune paralysis if the plasma concentration of immunoglobulins is below normal ranges, the replacement therapy with intravenous immunoglobulins?
*Consensus Rating* *: *Appropriate*; median score 7 (IQR 6–8); Disagreement: NO*
  5.3% voted in the 1–3 region, 26.3% voted in the 4–6 region, 68.4% voted in the 7–9 region

(3) How appropriate is, in patients with septic shock and suspected immune dysfunction/immune paralysis and with decision to use intravenous immunoglobulins, the use of a preparation including also IgM component?
*Consensus Rating* *: *Appropriate*; median score 8 (IQR 7-9); Disagreement: NO*
  5.3% voted in the 1–3 region, 5.3% voted in the 4–6 region, 89.4% voted in the 7–9 region

##### Other immunotherapeutic agents

1. How appropriate is, in patients with septic shock and suspected immune dysfunction/immune paralysis, and lymphopenia (< 600 cells/mcl) or low monocyte HLA-DR expression, the supportive therapy with GM-CSF?
*Consensus Rating:* Uncertain*; median score 5 (IQR 4-7); Disagreement: YES*
  21.1% voted in the 1–3 region, 52.6% voted in the 4–6 region, 26.3% voted in the 7–9 region
2. How appropriate is, in patients with septic shock and suspected immune dysfunction/immune paralysis, the use of other immunotherapeutic agents (i.e. IL7, IFN-gamma, Anti PD1-PDL1)? 
*Consensus Rating* *: *Uncertain*; median score 4 (IQR 3–5); Disagreement: YES*
  42.1% voted in the 1–3 region, 57.9% voted in the 4–6 region

##### Rationale for therapies

**Steroids**

*Septic shock:* Corticosteroids have been used as adjunctive therapy for septic shock for at least 40 years because of their potent anti-inflammatory activity and by considering their altered production during sepsis [22]. Steroids exert their anti-inflammatory activity by inhibiting leukocyte extravasation, function of macrophages and antigen-presenting cells, and production of TNF-alpha, interleukin-1 and nitric oxide. The incidence of adrenal dysfunction during septic shock has been estimated to be 50%, mainly due to complex derangements which include functional alterations in endocrine organs [23]. In the 1970s and the beginning 1980s, high-dose steroids (30 mg/kg methylprednisolone or 3–6 mg/kg dexamethasone) were used in septic patients. Thereafter, this approach was dismissed because of randomised clinical trials showing an increased risk of secondary infections, gastrointestinal bleeding and lack of improvement in overall survival [24, 25]. More recently, several studies have demonstrated that low doses of hydrocortisone (200–300 mg/day) improve haemodynamic and organ function with early weaning from vasoactive drugs, with minor adverse events [26–32]. Despite the strong pathophysiological rationale, the evidence for their benefit in terms of mortality reduction remains controversial (summary of evidence in the Supplementary material). In the last edition of the Surviving Sepsis Campaign Guidelines [4], administration of hydrocortisone at a dose of 200 mg per day is suggested in patients with septic shock if adequate fluid resuscitation and vasopressor therapy (norepinephrine or epinephrine ≥ 0.25 mcg/kg/min) are not able to restore haemodynamic stability after 4 h (weak recommendation with moderate quality of evidence). Moreover, several practical questions remain unanswered, such as the patient population that can achieve the best benefit, appropriate dose and method of administration (i.e. continuous infusion or refractory boluses), optimal duration of therapy and need for dose titration [4].

No study has specifically evaluated the effects of low-dose steroids in patients with septic shock and immune paralysis.

*Community-acquired pneumonia:* Community-acquired pneumonia (CAP) remains one of the main causes of death from infections in developed countries, although the survival rate has improved in the last decades [33]. Excessive production of pulmonary cytokines induced by pulmonary infection may cause a severe host inflammatory response, inducing pulmonary dysfunction and a higher risk of ICU admission and mortality [34]. Corticosteroids, with their potent anti-inflammatory activity, could therefore be effective, especially in patients with severe CAP (sCAP). Unfortunately, there are only a few randomised controlled trials on the use of corticosteroids in sCAP, with controversial results. Recent studies have demonstrated that IV steroids (hydrocortisone 200 mg, followed by 10 mg/h for 7 days or methylprednisolone 0.5 mg/kg in bolus 2/day for 5–7 days) may decrease treatment failure, duration of mechanical ventilation, ICU stay, mortality and complications such as ARDS and shock [35–39]. More recently, a multicentre RCT evaluating the use of low-dose methylprednisolone in severe CAP was performed in the USA in 586 ICU patients and failed to demonstrate a reduction in 60-day mortality even after sensitivity analysis [40]. Conversely, Dequin and colleagues published in March 2023 the CAPE COD trial [41], a multicenter double-blind RCT randomising 795 patients with severe CAP to receive intravenous hydrocortisone (200 mg daily by continuous infusion for either 4 or 7 days as determined by clinical improvement) or placebo. Patients with septic shock or influenza were excluded from this study. The study showed that hydrocortisone reduced the 28-day mortality without a higher rate of adverse events. In April 2023, the ERS/ESICM/ESCMID/ALAT guidelines for the management of severe CAP suggested the use of corticosteroids if shock is present (conditional recommendation, low quality of evidence). The authors also suggest that when corticosteroid therapy is considered, methylprednisolone (0.5 mg/kg every 12 h for 5 days) is a reasonable option [42]. Recently, a pairwise dose–response meta-analysis including 18 studies and 4661 patients [43] found that, despite the high heterogeneity of the included studies, treatment with corticosteroids was associated with a probable reduction in mortality only in patients with more severe CAP. Notably, the study showed a nonlinear dose–response relationship with mortality. In a specific subset of viral CAP due to influenza, two recent meta-analyses demonstrated that the use of corticosteroids increased mortality, ICU LOS and the rate of secondary infection in patients with influenza pneumonia, without affecting the duration of mechanical ventilation [44, 45].

*Bacterial meningitis*. Despite adequate antibiotic therapy and advances in supportive therapies, bacterial meningitis remains associated with high mortality and morbidity rates [46]. In particular, the risk of mortality and neurological sequelae in survivors is high, especially in patients with pneumococcal and Listeria monocytogenes meningitis [47, 48]. In the last year, it became clear that bacterial lysis due to antibiotic treatment and the subsequent inflammatory response played a pivotal role in the development of organ dysfunction [47]. Therefore, the early administration of steroids may be useful as an early adjunctive therapy [49]. A recent Cochrane review showed that early corticosteroid administration (usually dexamethasone 0.6 mg/kg) before or with the first dose of antibiotics is effective in reducing hearing loss and neurological sequelae, but not overall mortality, in adults and children with bacterial meningitis, at least in high-income countries [50]. The duration and long-term effects of corticosteroid therapy are important issues that remain unresolved.

In summary, the panellists agreed that in specific cases of refractory septic shock and severe hyperinflammatory response, the use of low-dose steroids may be warranted, although the optimal administration strategy remains unclear. The panellists concurred that suspending both low-dose steroid and vasopressor therapy was appropriate in this context. For severe community-acquired pneumonia, early use of steroids, such as methylprednisolone 40 mg/day or hydrocortisone 200 mg/day, may be considered, except when influenza is diagnosed. In the case of bacterial meningitis, the very early administration of steroids, either concurrently or prior to antibiotics, with dexamethasone 0.6 mg/kg/day or equivalent for 5–7 days, may be appropriate.

**Blood purification**

In recent years, the rationale for using blood purification techniques in sepsis has evolved from the concept of broad clearance of toxic humoral substances to the more selective removal of specific targets involved in the immune-inflammatory response. Initially, it was believed that lowering the plasma levels of pro-inflammatory mediators in the first phase of sepsis could be beneficial [51]. Subsequently, it was theorised that blood purification may play a role in immunomodulation by restoring the balance between pro- and anti-inflammatory response [52]. Furthermore, it has been suggested that the potential benefits of blood purification techniques might depend on cytokine tissue washout induced by a concentration gradient between plasma and tissue [53]. Despite the pathophysiological rational and the promising findings from animal models and initial clinical experiences, the evidence supporting blood purification in sepsis is controversial and for this reason the Surviving Sepsis Campaign Guidelines [4] suggested against the use of Polymyxin B heamadsorption and did not consider any other technique. The term blood purification encompasses various techniques, including high-volume haemofiltration, adsorption haemofiltration, high-cut-off membrane haemofiltration, plasma exchange and hybrid systems such as coupled plasma filtration adsorption. Among these, the panel decided to focus on the most used techniques: high-volume haemofiltration, extracorporeal cytokine hemadsorption, endotoxin hemadsorption and coupled plasma haemofiltration and absorption.

*High-volume haemofiltration*: High-volume haemofiltration (HVHF) is defined as continuous renal replacement treatment with volumes between 50 and 70 ml/kg/h or intermittent treatment with volumes of 100–120 ml/kg/h for 4–8 h [54, 55]. During sepsis, HVHF was supposed to improve the clearance of inflammatory mediators, and preliminary clinical studies have demonstrated that increasing doses of haemofiltration were associated with better patient outcomes [56, 57]. Unfortunately, the multicentre IVOIRE study showed no difference in 28-day mortality and haemodynamic variables in 140 patients with septic shock randomised to receive HVHF or standard haemofiltration [58]. Similarly, a single-centre RCT [59] on 280 patients with sepsis and acute kidney injury undergoing high-volume haemofiltration (50 mL/kg/h, HVHF) or extra-high-volume haemofiltration (85 mL/kg/h, EHVHF) showed no difference in mortality as well as in renal and other secondary outcomes between the two treatments. Meta-analyses [60, 61] also concluded that HVHF in comparison with standard renal replacement therapy does not provide any benefit in terms of survival rate, prevention or restoration of renal function, vasopressor-free days and incidence of adverse events.

*Extracorporeal cytokine hemadsorption*: In septic patients, extracorporeal cytokine hemadsorption is aimed at removing both pro- and anti-inflammatory cytokines from the blood. Animal studies have demonstrated that extracorporeal cytokine hemadsorption can reduce the levels of circulating mediators, such as TNF, IL-6 and myoglobin, which may reduce morbidity and organ damage in patients with a hyperinflammatory response and high levels of circulating cytokines [62–64]. In addition, it has been hypothesised that extracorporeal cytokine hemadsorption may exert the greatest benefit when initiated very early after sepsis occurrence [64]. Unfortunately, few low-quality studies have been published on the use of this technique in patients with sepsis. A multicentre RCT enrolling 97 patients with acute lung injury and septic shock showed that extracorporeal cytokine hemadsorption treatment was able to decrease serum IL-6 levels but without any effect on the PaO_2_/FiO_2_ ratio, organ dysfunction and mortality [65]. Similar results were obtained from the international registry on the use of extracorporeal cytokine hemadsorption in ICU patients, including 198 patients with sepsis [66], and from a prospective monocentric study in Germany on 20 patients with refractory septic shock receiving haemoperfusion with extracorporeal cytokine hemadsorption very early after shock occurrence [67].

*Endotoxin hemadsorption*: Owing to its ability to bind endotoxins, Polymyxin B was initially used as a parenteral drug to counteract the negative effects of endotoxaemia caused by gram-negative infections. Unfortunately, parenteral use has been rapidly abandoned owing to significant neurological and renal toxicity. Thereafter, the concept of using a cartridge with immobilised Polymyxin B (PMX-B) for extracorporeal haemoperfusion was proposed. In 2009, the Italian multicentre EUPHAS trial demonstrated in 64 patients with abdominal infections undergoing emergency surgery, that the early use of PMX-B haemoperfusion was associated with a reduction in the use of vasopressor drugs, improvement in SOFA score and 28-day mortality [68]. Conversely, in 2015, the French multicentre ABDOMIX trial did not detect any difference in mortality and organ dysfunction in 243 patients with septic shock and confirmed peritonitis randomised to endotoxin hemadsorption or placebo [69]. Similarly, a large retrospective observational study including 413 patients with septic shock and gram-negative bacterial infection demonstrated no difference in 28-day mortality with the early use of endotoxin hemadsorption [70]. This study was included in a systematic review and meta-analysis of 17 trials that outlined a correlation between patient severity and the effects of endotoxin hemadsorption, with a significant reduction in mortality in the intermediate- and high-risk groups, but not in the low-risk group [71]. The recently published multicentre EUPHRATES trial randomised 450 patients with refractory septic shock and high levels of endotoxin in the blood to receive standard treatment plus two endotoxin hemadsorption treatments (90–120 min) or sham within 24 h of enrolment. Endotoxin hemadsorption was not associated with a significant difference in mortality at 28 days in the entire patient sample or in the subgroup of patients with a multiple organ dysfunction score of > 9 [72]. A post hoc analysis of this trial showed that endotoxin hemadsorption seems to be effective in improving mortality and ventilator-free days in a specific population of patients with plasma endotoxin activity levels between 0.6 and 0.89 [73]. Further analysis, including data from a large observational trial [74] and the EUPHRATES trial, showed that abnormal coagulation and hyperlactatemia in septic patients with high endotoxin activity can be useful in identifying those who may benefit the most from PMX-HA [75]. Finally, a recent meta-analysis including 6 RCTs and 857 patients indicated with low grade of certainty that endotoxin hemadsorption did not result in any significant improvement in mortality and organ dysfunction in patients with sepsis and septic shock [76].

*Coupled plasma filtration adsorption (CPFA)*: CPFA is a hybrid technique that combines filtration with the separation of plasma from blood and absorption with plasma flow through a resin cartridge devoted to nonspecific adsorption of pro- and anti-inflammatory mediators and endotoxins. The body of evidence regarding the use of CPFA in patients with sepsis remains heterogeneous. The first clinical study [77] evaluated 20 patients with septic shock treated with CPFA and showed an improvement in the mean arterial pressure, cardiac index and PaO_2_/FiO_2_ ratio. The prospective multicentre study COMPACT randomised 192 patients with septic shock to standard therapy plus CPFA or placebo and demonstrated that CPFA improved neither mortality nor other clinical outcomes [78]. Other retrospective analyses demonstrated positive effects of CPFA on haemodynamic variables with different dose- and time-related efficacy [79, 80]. The COMPACT-2 trial, which aimed to assess whether high doses of CPFA may improve mortality in patients with septic shock, was prematurely stopped after 103 patients (out of 350) by the Data Safety Monitoring Board because of an excess of mortality in patients treated with CPFA [81].

In summary, the use of HVHF or CPFA in individuals with septic shock and hyperinflammatory response is deemed inadvisable. Furthermore, the efficacy of endotoxin hemadsorption and extracorporeal cytokine hemadsorption haemoperfusion remains unclear in patients with septic shock and hyperinflammatory response.

It is important to remind that several extracorporeal techniques, as for instance HVHF, CPFA and extracorporeal cytokine hemadsorption, may favourite the removal of antibiotics, resulting in an unpredictable reduction of antimicrobial plasma levels. To prevent underexposure to antibiotics, particularly in patients with infections caused by difficult-to-treat microorganisms, the panel recommends increasing antibiotic dosages and, when possible, assessing antibiotic plasma concentrations during or after treatment.

**Immunoglobulins**

Endogenous immunoglobulins (Igs) constitute an essential component of the immune response with complex and not fully understood mechanisms that interact with both innate and adaptive immunity. Igs mediate and participate in the activation of pro-inflammatory responses and simultaneously exert anti-inflammatory activity via cytokine neutralisation, upregulation of receptors with inhibitory activities, complement cascade inhibition and modulation of dendritic cells activity [82, 83]. In patients with sepsis, low levels of circulating immunoglobulins are common and associated with worse outcomes. Notably, it has been shown that IgM plasma concentration in the first week after septic shock occurrence was considerably higher in survivors than in non-survivors [84, 85]. These observations led to the use of intravenous polyclonal Ig preparation (IVIg) as adjunctive therapy in adults and children with sepsis and septic shock in the last 25 years. Unfortunately, data available so far are not conclusive and clear evidence for benefit in sepsis is lacking. Several meta-analyses [86–89] published in the last 10 years with the inclusion of approximately 20 randomised controlled trials on more than 2000 patients showed that the use of Ig preparations in patients with sepsis seems to provide a significant reduction in short-term mortality; however, the low quality of the studies and the important grade of heterogeneity hinder any robust conclusion for efficacy. For the above reasons, and principally considering the results of the large SBITS trial [90], the last edition of the SSC Guidelines advised against the use of Ig preparations with a weak recommendation and a low level of evidence [91]. The SBITS trial [90] investigated the efficacy of a 2-day treatment with IgG polyclonal immunoglobulins in 647 patients with sepsis and found no difference between treated and non-treated patients in 28-day survival and length of mechanical ventilation with only a slight improvement in ICU survival. It is noteworthy that this study enrolled patients in the early 1990s (more than a decade before the publication), when the definitions and knowledge of sepsis management widely differed from today and, thereby, the inclusion criteria and treatments provided are highly questionable. Ongoing trials will better clarify the potential efficacy and which patients can benefit the most, the appropriate dose and time for adjunctive therapy with IVIg in sepsis. Meanwhile, as for other adjunctive therapies, pathophysiological considerations combined with clinical experience and literature data may guide the consideration of this therapy in specific clinical scenarios.

*Hyperinflammatory response*: In the first scenario considered in our consensus process (i.e. patients with abrupt and dysregulated hyperinflammatory responses), the rationale for IVIg therapy is based on the well-known effect of Igs as strong scavengers of pathogens, toxins and cytokines. A multicentre RCT performed in Sweden, Norway, Finland and the Netherlands evaluated the efficacy and safety of high-dose polyclonal IgG administration (standard preparation) as an adjunctive treatment for streptococcal toxic shock syndrome (STSS), which is a perfect example of a patient with a hyperinflammatory response [92]. Although the trial was prematurely interrupted after the inclusion of only 21 patients, the 28-day mortality and shock reversal time were lower in the patients treated with IVIg. A subsequent registry study of 67 patients with a diagnosis of STSS [93] showed that patients aged < 80 years had a significantly higher survival rate when treated with IVIg. Unfortunately, other trials have failed to confirm the benefits of IVIg therapy in patients with severe STI. The INSTINCT trial did not report any difference in 28-day mortality in 100 patients with necrotising STI randomised to a 3-day treatment with standard IVIg or placebo [94]. Similarly, a retrospective case–control study of 325 patients with necrotising fasciitis and septic shock who underwent surgical debridement showed no effect of standard IVIg therapy on hospital mortality and hospital stay [95]. In patients with severe community-acquired pneumonia, a post hoc analysis of the recent CIGMA trial highlighted that the use of a novel preparation of polyclonal immunoglobulins enriched with IgM reduced mortality only in the subgroup of patients with a hyperinflammatory phenotype assessed by C-reactive protein and procalcitonin [96]. Moreover, a recent study of 111 patients with meningococcal invasive disease indicated that early adjuvant therapy with an IgM-enriched preparation seems to improve the outcome with a reduction in mortality and permanent neurological sequelae [97].

*Immune paralysis*: In patients with immune dysfunction and persistent immune paralysis, the rationale for using IVIg is based on the pleiotropic activities of immunoglobulins, particularly IgM, on immune cell networks, with evidence of anti-apoptotic and direct anti-inflammatory properties [89]. Persistent infections by opportunistic bacteria are considered a pathognomonic sign of severe impairment of the immune response [98]. Two retrospective studies including approximately 300 patients with sepsis due to MDR infections admitted to Greek and Italian ICUs showed that adjunctive therapy with IVIg enriched in IgM provided a consistent reduction in mortality of approximately 20% [99, 100]. However, in patients with severe immune system failure, such as neutropenic patients with haematological malignancies, a multicentre RCT failed to demonstrate any benefit in terms of survival rate by using IVIg enriched in IgM therapy during sepsis or septic shock [101].

Standard preparations of IVIg contain polyclonal class-G immunoglobulins, with only traces of IgA and IgM. The key role of IgM in innate and adaptive immune processes [83] has led to the development of an IgM-enriched preparation that better reproduces the physiological antibody concentration in the plasma. Although some literature data seem to indicate that in septic patients, IgM-enriched preparation might be more effective than standard polyclonal IVIg containing only IgG [86–88], the low quality and high heterogeneity of evidence led the experts of the SSC guidelines to suggest against the routine use of these preparations (weak recommendation, low quality of evidence) [4].

Concerning the appropriate time for starting IVIg therapy, a retrospective analysis of 355 patients with sepsis and septic shock demonstrated that delayed administration of IgM preparation from admission to the ICU was associated with an increased risk of ICU mortality independent of SAPS II [102].

In summary, although the utility of intravenous immunoglobulins in treating septic shock remains unclear, the early administration of a formulation that includes an IgM component may be advisable in selected patients, such as those with septic shock and hyperinflammatory response due to toxin-related syndromes (e.g. invasive meningococcal diseases, pneumococcal or meningococcal Purpura fulminans, NSTI/TSST and PVL necrotising pneumonia).

In patients with septic shock and suspected immune dysfunction/immune paralysis, if the plasma concentration of Ig is below the normal range, the use of IVIg, including IgM, may be useful in preventing secondary infections and supporting antibiotic therapy for difficult-to-treat microorganisms.

##### Rationale for granulocyte–macrophage colony-stimulating factor

Granulocyte–macrophage colony-stimulating factor (GM-CSF) stimulates the proliferation and maturation of immune cells, enhancing the antimicrobial host response by increasing the motility, phagocytic activity and respiratory burst of neutrophils and monocytes/macrophages. Moreover, GM-CSF seems to increase mHLA-DR expression and reverse the long-lasting monocyte deactivation that occurs frequently in sepsis [103]. Beyond its normal use in chemotherapy-induced febrile neutropenia [104], GM-CSF has been shown to have promising effects in non-neutropenic neonatal and adult sepsis [105–107]. Unfortunately, many of the published studies have significant limitations due to the low number and heterogeneity of the population and the variability in dosage, chemical formulations and administration routes. Moreover, the effects of the timing of administration or patient stratification on immunological status have never been explored. A meta-analysis of 12 RCTS and 2380 patients evaluated the effects of G-CSF or GM-CSF therapy in non-neutropenic patients with sepsis [107]. The analysis showed no significant difference in 28-day mortality or hospital mortality when G-CSF or GM-CSF were compared with placebo. Nevertheless, although the data were available only in four trials, the administration of G-CSF or GM-CSF significantly increased the reversal from infection without any adverse events. Among the RCTs considered in the meta-analysis, the trial conducted by Meisel et al. [108] was remarkable because it included only patients with immunosuppression (e.g. low levels of HLA-DR on monocytes) after sepsis. The study was not powered to assess differences in mortality but aimed to evaluate the effects of GM-CSF on the immune response. As expected, a significant restoration of monocyte HLA-DR expression and cytokine production was observed, with a trend toward favourable outcomes in patients treated with GM-CSF for up to 9 days. After the publication of a meta-analysis [107], a RCT [109] explored the effects of GM-CSF in 130 patients with ALI and ARDS caused mainly by pneumonia. No differences were found in ventilator-free days (primary outcome), 28-day mortality, or organ failure duration. In 2018, a phase IIa randomised, placebo-controlled clinical trial was conducted in critically ill patients with impaired neutrophil phagocytosis, randomised to either subcutaneous GM-CSF (3 μg/kg/day) or placebo [110]. Notably, less than 50% of the included patients had sepsis. In the GM-CSF group, the authors found a higher proportion of patients with ≥ 50% neutrophil phagocytosis on day 2 and significantly higher monocyte HLA-DR expression, and the most common adverse event associated with GM-CSF was fever. Recently, a randomised, double-blind, placebo-controlled clinical trial was conducted on 66 sepsis patients with ARDS of extrapulmonary origin who received intravenous recombinant GM-CSF or placebo [111]. The study analysed the levels of inflammatory cells, HLA-DR, HMGB-1, TNF-α, IL-6 and GM-CSF in both blood and bronchoalveolar lavage fluid. Treatment group significantly enhanced PaO_2_/FiO_2_ ratio, without any benefit in ventilator-associated pneumonia incidence and 28-day mortality. Moreover, the experimental group demonstrated an improvement in the inflammatory reaction in the lungs without affecting the inflammatory levels in the blood. Another open-label RCT evaluated the effects of combining intravenous GM-CSF with Meropenem in 131 cirrhosis patients with difficult-to-treat spontaneous bacterial peritonitis (SBP) [112]. The group treated with GM-CSF had higher SBP early response and SBP resolution rates than the group treated with meropenem alone. Moreover, the GM-CSF group had a lower incidence of pneumonia, acute kidney injury and other secondary infections.

The panel deemed it inappropriate to administer GM-CSF to individuals experiencing septic shock who presented with a severe hyperinflammatory response. Furthermore, the panel was uncertain about the advantages of using GM-CSF in individuals with septic shock and potential immune dysfunction or immune paralysis, in addition to lymphopenia (a count of less than 600 cells/µL) or low monocyte HLA-DR expression.

Other immune therapies with drugs aimed at blocking the effect of mediators or signalling molecules have been advocated as possible adjunctive treatments in patients with sepsis and impaired immune response [113]. Indeed, several immunotherapeutic agents, including recombinant interleukin-7 (IL-7), programmed cell death 1 (PD1)- or programmed cell death 1 ligand (PDL1)-specific antibodies and recombinant interferon-gamma (IFN-γ), have shown promising results in reversing the immunosuppressive phase of sepsis [21].

##### Rationale for other immunotherapeutic agents (IL-7, AntiPD1-PDL1, IFN-g)

IL-7, which is produced by bone marrow and thymus cells, is an indispensable cytokine for the growth, differentiation and effector functions of T cells. Recombinant human (rh)IL-7 has been proposed as an immune-enhancing agent in patients with lymphopenia, cancer and progressive multifocal leucoencephalopathy. Several preclinical studies have shown that rhIL-7 reduces T-cell apoptosis, restores IFN-γ production and enhances T-lymphocyte function in patients with sepsis [114–117]. A prospective double-blind, placebo-controlled pilot RCT in patients with septic shock and severe lymphopenia showed that recombinant human IL-7 (CYT107) was well tolerated without evidence of inducing a cytokine storm or worsening inflammation or organ dysfunction. Notably, it caused a 3- to fourfold increase in absolute lymphocyte counts and circulating CD4 + and CD8 + T cells that persisted for weeks after drug administration [114]. IL7 also demonstrated positive effects in enhancing the immune response during anti-PD1 treatment in cancer, suggesting a possible combination therapy in patients with sepsis [115–117]. A recent double-blind, placebo-controlled trial aimed to evaluate the effect of recombinant human IL-7 (CYT107) in twenty-one patients with septic-induced lymphopenia [118]. Prior to study enrolment, patients had to have persistent lymphopenia, defined as an absolute lymphocyte count of ≤ 900 cells/mm [3] within 48 h after the diagnosis of sepsis. Although the study drug seemed to reverse lymphopenia, the study was halted early because three of the 15 patients receiving intravenous CYT107 developed fever and respiratory distress approximately 5–8 h after drug administration.

PD1 receptor system represents a potent immunoregulatory pathway that negatively controls the immune response. This system consists of PD1 and its two ligands (PD-L1 and PD-L2). Several observational studies have described the increased expression of PD1-related molecules in circulating immune cells in patients with sepsis with immune dysfunction and negative outcomes [119]. Furthermore, ex vivo studies have shown that blockade of the PD1/PD-L 1 pathway is capable of limiting and restoring immune dysfunction associated with sepsis [120]. A phase 1 clinical study on the treatment of sepsis with nivolumab (an anti-PD1 blocking monoclonal antibody) is ongoing (NCT02960854), but so far, only a few reports are available on the use of this therapy in patients with sepsis [121].

IFN-γ is a prototypical type 1 helper T-cell cytokine and a major activator of monocytes with increasing antigen-presentation capacity and LPS-induced production of cytokines. The beneficial effect of IFN-ɣ-on monocyte deactivation in patients with sepsis was first described in 1997 in a limited open-label study, and its use in severely infected patients has only been reported in a few clinical cases [122]. Its use seems to improve immune functions, including an increase in monocyte HLA-DR expression, as well as the outcome and immune dysfunction in invasive fungal infections [123]. In a recent multicentre, placebo‑controlled trial, 109 critically ill patients with one or more acute organ failures and undergoing mechanical ventilation were randomised to receive interferon γ ‑1b or placebo [124]. Unfortunately, treatment with interferon did not significantly reduce the incidence of hospital-acquired pneumonia or mortality on day 28. Furthermore, the trial was discontinued early because of safety concerns.

Our panel was deemed inappropriate for the use of additional immunotherapeutic agents, including IL7, antiPD1-PD-L1 and IFN-γ, in patients with septic shock and severe hyperinflammatory response, as well as in those with septic shock and suspected immune dysfunction or immune paralysis.

### Supplementary Information

**Supplementary Material 1**.

## Data Availability

Data sharing is not applicable to this article, as no datasets were generated or analysed during the current study.

## Abbreviations

ICU: Intensive care unit
IL: Interleukin
Anti-PD: Antibodies targeting programmed cell death protein
PDL: Programmed cell death ligand protein
TNF: Tumour necrosis factor alpha
IFN: Interferon
CPFA: Coupled plasma filtration absorption
NSTI: Necrotizing soft tissue infection
TSST: Toxic shock syndrome toxin
PVL: Panton–Valentine leukocidin
Ig: Immunoglobulin
GM-CSF: Granulocyte monocyte colony-stimulating factor
G-CSF: Granulocyte-colony-stimulating factor
HLA-DR: Human Leukocyte Antigen – DR isotype
TGF: Transforming growth factor
Spp: Species
CAP: Community-acquired pneumonia
LOS: Length of hospital stay
HVHF: High-volume haemofiltration
EHVHF: Extra-high-volume haemofiltration
RCT: Randomised controlled trial
PaO2: Arterial artial pressure of oxygen
FiO2: Fraction of inspired oxygen
PMX: Polimixin
SOFA: Sequential organ failure assessment
IV: Intravenous
MDR: Multidrug resistant
SSC: Surviving Sepsis Campaign
HMGB-1: High-mobility group box 1
SBP: Spontaneous bacterial peritonitis
ALC: Absolute lymphocyte count
