# Supplementary material for: Adjunctive immunotherapeutic agents in patients with sepsis and septic shock: a multidisciplinary consensus of 23
Source: J Anesth Analg Crit Care. 2024 Apr 30;4:28. doi: 10.1186/s44158-024-00165-3 (PMC11059820; doi:10.1186/s44158-024-00165-3)
Supplement: Supplementary file 1 — Supplementary Material 1. [file 44158_2024_165_MOESM1_ESM.docx]

**SUPPLEMENTAL MATERIAL**

**1) Literature systematic review**

SEARCH METHODS

electronic databases or evidence source where the search will be performed

- PubMed, EMBASE, Cochrane

- Manual search of the retrieved full text

Time periods searched Publication year (2000-2022)

Search terms used.

Sepsis, Septic Shock, meningococcal disease, toxic shock syndrome, Adjunctive therapies, immune-paralysis, Immunomodulatory therapy, immunotherapy, corticosteroids, Immunoglobulins, IgM, Granulocyte/Monocyte Colony Stimulating Factor, Blood Purification Techniques, polymyxin B, hemoperfusion, plasma-apheresis, Interferon gamma, IL7-IL15, AntiPD1.

EVIDENCE SELECTION CRITERIA

Papers will be included if the following will apply:

Target population

- Patients with severe sepsis or septic shock are clearly identificable in outcome results (e.g. at least in a subgroup analysis)

Study design

- Randomised controlled trials

- Longitudinal studies (retrospective or prospective)

Comparisons (if relevant)

Studies in which at least one treatment arm includes the adjunctive therapy of interest

Outcomes

At least one of the following outcome is reported, for the group treated with the AT of interest: Overall mortality, ICU mortality, Hospital mortality, duration of shock, Duration of ICU stay, Duration of hospital stay, Duration of mechanical ventilation, Duration of inotrope support, Reinfection

Language (if relevant)

- Study results are available in English, Italian, Spanish, French

**2) Articles selection flow-chart**
